# Surveillance cultures of samples obtained from biopsy channels and automated endoscope reprocessors after high-level disinfection of gastrointestinal endoscopes

**DOI:** 10.1186/1471-230X-12-120

**Published:** 2012-09-03

**Authors:** King-Wah Chiu, Ming-Chao Tsai, Keng-Liang Wu, Yi-Chun Chiu, Ming-Tzung Lin, Tsung-Hui Hu

**Affiliations:** 1 Division of Gastroenterology and Hepatology, Department of Internal Medicine, Kaohsiung Chang Gung Memorial Hospital, Chang Gung University College of Medicine, 123 Ta-Pei Road, Niao-Sung District, Kaohsiung, 833, Taiwan

**Keywords:** Surveillance culture monitoring, Gastrointestinal scope, Automated endoscope reprocessor, High-level disinfection reprocessing

## Abstract

**Background:**

The instrument channels of gastrointestinal (GI) endoscopes may be heavily contaminated with bacteria even after high-level disinfection (HLD). The British Society of Gastroenterology guidelines emphasize the benefits of manually brushing endoscope channels and using automated endoscope reprocessors (AERs) for disinfecting endoscopes. In this study, we aimed to assess the effectiveness of decontamination using reprocessors after HLD by comparing the cultured samples obtained from biopsy channels (BCs) of GI endoscopes and the internal surfaces of AERs.

**Methods:**

We conducted a 5-year prospective study. Every month random consecutive sampling was carried out after a complete reprocessing cycle; 420 rinse and swabs samples were collected from BCs and internal surface of AERs, respectively. Of the 420 rinse samples collected from the BC of the GI endoscopes, 300 were obtained from the BCs of gastroscopes and 120 from BCs of colonoscopes. Samples were collected by flushing the BCs with sterile distilled water, and swabbing the residual water from the AERs after reprocessing. These samples were cultured to detect the presence of aerobic and anaerobic bacteria and mycobacteria.

**Results:**

The number of culture-positive samples obtained from BCs (13.6%, 57/420) was significantly higher than that obtained from AERs (1.7%, 7/420). In addition, the number of culture-positive samples obtained from the BCs of gastroscopes (10.7%, 32/300) and colonoscopes (20.8%, 25/120) were significantly higher than that obtained from AER reprocess to gastroscopes (2.0%, 6/300) and AER reprocess to colonoscopes (0.8%, 1/120).

**Conclusions:**

Culturing rinse samples obtained from BCs provides a better indication of the effectiveness of the decontamination of GI endoscopes after HLD than culturing the swab samples obtained from the inner surfaces of AERs as the swab samples only indicate whether the AERs are free from microbial contamination or not.

## Background

Flexible endoscopes are complex reusable instruments, and according to the British Society of Gastroenterology guidelines (February 2008) for decontamination of equipment used for gastrointestinal (GI) endoscopy, special care must be taken when decontaminating endoscopes. In addition to the external surfaces of GI endoscopes, their internal channels for air and water aspiration and other accessories are exposed to body fluids and other contaminants [1]. In our experience, instrument contamination can occur even after a complete reprocessing cycle using high-level disinfection (HLD). For example, a relief valve in the water supply system of an automated endoscope reprocessor (AER) may be damaged and disconnected [2]. Therefore, surveillance culture may help in monitoring the effectiveness of GI endoscope disinfection. The first step in decontamination of the GI endoscopes is thorough manual cleaning using a compatible enzymatic detergent; all accessible channels should be brushed with the detergent and then flushed with sterile distilled water, before automatic disinfection using an AER. We conducted this prospective study to assess the effectiveness of a complete reprocessing cycle in decontaminating GI endoscopes. To this end, we performed culture studies on the samples obtained from biopsy channels (BCs) of the upper (gastroscope) and lower (colonoscope) of the GI endoscopes and from the internal surfaces of the AERs.

## Methods

**Figure 1:**
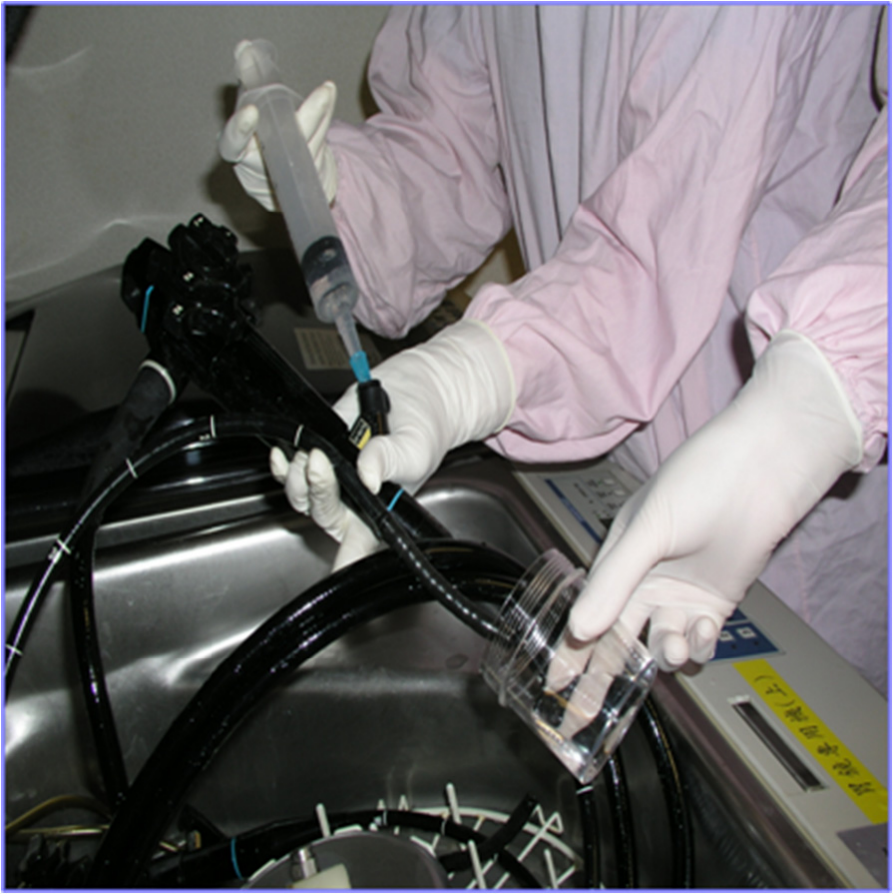
**Cultures of rinse samples from GI endoscopes.** Samples were obtained by flushing 50 ml sterile distilled water through the biopsy channel under highly aseptic conditions. The flushed fluid was collected in a sterile container and plated onto blood agar and MacConkey agar plates and inoculated into Lowenstein–Jensen medium.

**Figure 2:**
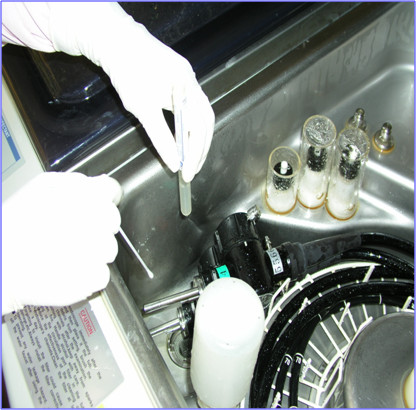
**Cultures of swabs of residual water from automated endoscope reprocessors (AERs).** Immediately after completion of a high-level disinfection cycle, residual water from the inner surfaces of the AERs was collected using swabs under aseptic conditions.

This 5-year (February 2006–January 2011) prospective study was performed at Chang Gung Memorial Hospital, Kaohsiung Medical Center, Taiwan. Every month, we took random consecutive swabs and BC samples from 7 AERs (including 5 reprocess to gastroscopes and 2 reprocess tocolonoscopes). A total of 420 samples were obtained by rinsing the BCs of gastroscopes and colonoscopes, and 420 swab samples of residual water were collected from the internal surfaces of the AERs after a complete reprocessing cycle. Of these 840 samples, 600 were from gastroscopes (300 samples each from BCs and 300 from AERs reprocess to gastroscopes) and 240 from colonoscopes (120 samples each from BCs and 120 from AERs reprocess to colonoscopes). The rinse samples were obtained by flushing the BCs with 50 ml sterile distilled water under highly aseptic conditions (Figure 1). The distilled water was contained in an aseptic vial (20 mL/vial) manufactured for medical use. The residual water, after flushing, was collected in a sterile container and plated on blood agar, MacConkey agar, and Lowenstein–Jensen medium. We collected swab samples of the residual water from the internal surfaces of the AERs after a complete reprocessing cycle (Figure 2). Swabs were placed in liquid thioglycollate broth for primary culture. Sampling of the BC rinsing fluid and swab cultures from the internal surface of the AERs were performed simultaneously. The samples were incubated at 37°C and presence of aerobic and anaerobic bacteria and *Mycobacterium tuberculosis* was determined after 24 h, 48 h, and 6 weeks. The growth of all strains was determined by counting the colony forming units (CFU) isolated from the samples by plate count method and incubated for 30 h at an optimal temperature. Samples with plate counts greater than 10^3^ CFU/mL were defined as culture-positive samples. The instruments examined were reported as being culture-positive or culture-negative. If a GI endoscope determined to be culture-positive, that specific GI endoscope was no longer used. The soaking duration was increased to 25 min. Adequate precleaning manual brushing of the biopsy channel at least 3–5 times was carried by nurses. Only culture-negative GI endoscope is returned to clinical use [2]. GI endoscope decontamination was performed in accordance with the guidelines set by the European Society of Gastrointestinal Endoscopy [3]. The endoscopes were cleaned manually by trained GI nurses by brushing and rinsing with tap water and an enzymatic detergent. Manual cleaning was followed by decontamination by a trained healthcare technician using an AER (EW-30, Aizu Olympus Co. Ltd, Fukushima, Japan). The liquid disinfectant used was 2.4% alkaline glutaraldehyde, which was stored at 15–30°C and replaced every 2 weeks; the endoscopes were soaked in the disinfectant for 20 min. The disinfectant was monitored by the endoscopic nurse (Miss Ching-Yin Huang) every morning using test strips, and was discarded within 14 days if the concentration dropped below the minimum effective concentration (MEC). If the GI endoscopes were found to be culture-positive, the soaking duration was increased to 25 min. The disinfectant solution was forced into the endoscopes until the internal channels were filled. Subsequently, the endoscopes were flushed with sterile distilled water and dried with forced air (high pressure forced air with a 25 L/min airflow and 60 psi pressure to ensure more thorough drying of the internal channels of the GI endoscope after a complete HLD reprocessing cycle).

The X^2^ test was used for statistical analysis of independent and paired samples. Statistical analyses were performed using the Statistical Package for the Social Sciences (SPSS) version 14.0 for Windows (Chicago, IL, USA). A p value <0.05 was considered statistically significant.

## Results

A total of 13.6% (57/420) BC samples and 1.7% (7/420) AER samples were found to be culture-positive. We observed a statistically significant difference in the number of samples collected from the BCs and AERs that were positive for aerobic bacteria (p < 0.0001) (Table 1). 10.7% [32/300] of the samples collected from the BC of gastroscopes and 20.8% [25/120] samples from the colonoscopes were found to be culture-positive and were significantly higher than those collected from the AERs reprocess to gastroscope (2.0% [6/300]) and the AERs reprocess to colonoscope (0.8% [1/120]) There was a significant difference in the contamination rate of the endoscopes used for the upper and lower GI tracts (p = 0.00585) (Table 1). Of the samples collected from the AERs reprocess to the gastroscope and colonoscope, 2.0% (6/300) and 0.8% (1/120), respectively, were found to be culture-positive and there was no significant difference in the contamination rate between the AERs reprocess to the upper and lower GI tract (p > 0.05). Analysis of the 57 culture-positive samples obtained from BCs revealed that 69 species of bacteria had formed colonies; 89.5% (51/57) of samples showed colonization by a single species and 10.5% (6/57) colonization by multiple species. The number of samples colonized by a single species of culture-positive bacteria (93.75% [30/32] samples from BC of gastroscopes and 84.0% [21/25] from BC of colonoscopes) were greater than those colonized by multiple species (6.25% [2/32] of gastroscope BCs and 16.0% [4/25] of colonoscope BCs) (Table 2). No statistically significant difference was observed in the distribution of bacterial flora between samples obtained from gastroscopes and colonoscopes. Cultures of all 7 swab samples collected from AERs (6 from gastroscope AERs and 1 from a colonoscope AER) showed colonization by a single species. None of the cultures were found to be aerobic or anaerobic bacteria or *M*. *tuberculosis*. Of the 64 culture-positive samples, 95.3% (61/64) were positive for aerobic bacteria and only 4.7% (3/64) were positive for anaerobic bacteria (Table 3). None of the culture-positive swab samples obtained from the AERs after a complete reprocessing cycle revealed the presence of anaerobic bacteria. Most of the aerobic bacteria (68.4% [39/57]) present in the samples collected from the BCs of gastroscopes (75.0% [24/32]) and colonoscopes (60.0% [15/25]) were glucose non fermenting Gram-negative (GNGN) bacteria; *Escherichia coli* was only found in culture-positive samples obtained from the colonoscope BCs (10.5% [6/57]) (Table 3). A higher number of culture-positive samples were colonized by a single bacterial species than by multiple species. Similar findings were observed for the samples obtained from gastroscopes and colonoscopes after a complete reprocessing cycle. Yeast-like bacteria were present in the swab samples obtained from the AERs reprocess to the ggastroscope and colonoscope, but were absent from the samples obtained from the BCs. In addition, specific bacteria were sporadically found in some samples obtained from the gastroscopes and colonoscopes.

**Table 1:** The number of positive bacterial cultures obtained from rinse samples from biopsy channels of gastroscopes and colonoscopes and from swab samples obtained from automated endoscope reprocessors used for the decontamination of gastroscopes and colonoscopes

**Category**	**BC No. (%)**	**AER No. (%)**	**p value**
Gastroscope, n = 300	32 (10.7)*	6 (2.0)	<0.0001
Colonoscope, n = 120	25 (20.8)*	1 (0.8)	<0.0001
Total, n = 420	57 (13.6)	7 (1.7)	<0.0001

BC, biopsy channel; AER, automated endoscope reprocessor.

Pearson chi-squared test showed that the number of positive bacterial cultures from the samples obtained from BCs was significantly higher than that from the samples obtained from the AERs (p < 0.0001).

* Fisher’s exact test showed a statistically significant difference between the number of positive bacterial cultures from samples obtained from the BCs of gastroscopes and those obtained from colonoscopes (p = 0.00585).

**Table 2:** Bacterial flora of the culture-positive rinse samples obtained from the biopsy channels of gastroscopes and colonoscopes after reprocessing

**Category**	**Single species No. (%)**	**Multiple species No. (%)**
Gastroscope, n = 32	30 (93.75)	2 (6.25)
Colonoscope, n = 25	21 (84.0)	4 (16.0)
Total, n = 57	51 (89.5)	6 (10.5)

**Table 3:** Organisms identified from cultures of samples collected from the biopsy channels of gastroscopes and colonoscopes and from swab cultures of samples obtained from automated endoscope reprocessors

**Category Organism**	**Gastroscope**		**Colonoscope**		**Total**
	**Single species**	**Multiple species**	**Single species**	**Multiple species**	
Organisms in the cultures of rinse samples obtained from biopsy channels					
GNGN bacteria**	22	2^a, b^	15		39
Yeast-like bacteria	1		1		2
*Klebsiella pneumoniae*	1			1^c^	2
*Acinetobacter baumanii*		1^b^		1^f^	2
*Enterococcus* spp.		1^b^		2^c, d^	3
*Comamonas testosteroni*	2				2
*Chryseobacterium indologenes*	1				1
*Sphingomonas paucimobilis*	1				1
*Pseudomonas putida*	1				1
Viridans *Streptococcus*		1^a^			1
*Stomatococcus* spp.		1^a^			1
*Prevotella bivia*	1*				1
*Escherichia coli*			3	3^c, d, f^	6
*Pseudomonas aeruginosa*			1		1
*Enterococcus faecium*				1^c^	1
*Bacteroides fragilis*				1^e^*	1
*Bacteroides vulgatus*				1^e^*	1
*Bacteroides distasonis*				1^e^*	1
*Clostridium perfringens*			1*		1
*Proteus mirabilis*				1^d^	1
Total number of bacteria	36		33		69
Total number of positive cultures	32		25		57
Organisms in swab cultures of samples obtained from automated endoscope reprocessors#					
GNGN bacteria**	2		1		3
Yeast-like bacteria	2				2
*Moraxella osloensis*	1				1
*Candida glabrata*	1				1
Total number of positive cultures	6		1		7

*Anaerobic bacteria; the rest were cultures of aerobic bacteria.

**GNGN bacteria: Glucose non fermenting Gram-negative bacteria.

a, b: Cultures of samples obtained from gastroscopes colonized by multiple species.

c, d, e, f: Cultures obtained from colonoscopes colonized by multiple species.

#: Chiu et al. World J Gastroenterol. 2012 Apr 14;18(14):1660–3.

## Discussion

AERs subjected to HLD are widely used for the decontamination of endoscopes. The guidelines of the British Society of Gastroenterology emphasize the benefits of manual brushing of endoscope channels in addition to automated decontamination [1]. We aimed to determine whether adequate decontamination of AERs and GI endoscopes was achieved after complete reprocessing. This 5-year prospective study showed that the number of culture-positive samples obtained from the BCs (13.6% [57/420]) of GI endoscopes was significantly higher than those obtained from swab samples taken from the internal surfaces of AERs (1.7% [7/420]) after HLD (p < 0.0001). This finding may be due to the fact that the structure of an endoscope is more complicated than that of an AER. GI endoscopes are complex reusable instruments that require special care during decontamination. Contamination of the AER samples may have been underestimated because the structures of the GI endoscopes and that of the AERs differ. Endoscopes are complicated instruments with multiple internal channels (air, water, suction, and biopsy channels) with many dead air spaces and complete disinfection is difficult to achieve. BCs are easily contaminated by patient body fluids, blood, or tissue. The finding of a higher contamination rate in BCs compared with AERs was also true for the rinse samples obtained from the BCs and the swab samples obtained from the AERs reprocess to the gastroscope (10.7% [32/300] *vs*. 2.0% [6/300]; p < 0.0001) and colonoscope (20.8% [25/120] *vs*. 0.8% [1/120]; p < 0.0001) AERs. Most updated guidelines emphasize adequate decontamination of endoscopes [1,4-7]. There was greater contamination in 160 m long colonoscopes than in the 100 cm long colonoscopies (p = 0.00585); decontamination of a gastroscope was easily achieved after HLD reprocessing. Endoscopes inserted via the anal route are reportedly more contaminated than those inserted orally, and a 200 cm long endoscope is more difficult to decontaminate than a 100 cm long endoscope [8].The surveillance resulting consensus document defines invasive procedures in detail and provides endoscopists with practical advice on how to avoid contamination of BCs by lymphoid tissue during endoscopic biopsy and other therapeutic procedures. BCs are the most complicated components of GI endoscopes and they are difficult to disinfect when they become contaminated with highly infectious material, such as that associated with fine needle puncture biopsy of lymphoid tissue. Sheathed biopsy forceps may be introduced to improve the safety of biopsies in at-risk individuals and to avoid BC contamination of GI endoscopes. This change is intended to address concerns about a new variant of Creutzfeldt-Jakob disease (vCJD) and is emphasized by the British Society of Gastroenterology guidelines (February 2008). The length of the endoscope is an important factor that adds to the difficulty of disinfection [8]. In addition, adequate manual pre-cleaning of long endoscopes with complicated internal channels, such as BCs, is difficult. Therefore, contamination of AERs may be a consequence of their design and is not necessarily a result or by-product of the design of GI endoscopes or the quality of reprocessing. The inner surfaces of the BCs may not be sufficiently decontaminated even after a complete reprocessing cycle. Regular monitoring of reprocessing is important for ensuring quality and patient safety [9]. Our results suggest that culturing samples obtained by rinsing the BCs is one of the best methods for performing regular monitoring. A review of recent reports highlights cost concerns along with the importance of monitoring the microbial content of rinse samples from BCs [10]. Regular monitoring of GI endoscopes and HLD decontamination is important for ensuring patient safety and should not be reserved for infectious disease outbreaks. Our data indicates that the culturing of rinse samples from BCs of GI endoscopes (compared with AER samples) and from colonoscopes (compared with gastroscopes) is the best indicator of the effectiveness of the decontamination process.

An interesting finding with regard to the samples obtained from the BCs of gastroscopes (93.75% [30/32]) and colonoscopes (84.0% [21/25]) after reprocessing was that most samples were colonized by a single bacterial species. Sporadic contamination by multiple species was observed in only 2 gastroscope (6.25% [2/32]) and 4 colonoscope (16.0% [4/25]) samples. This incidence did not differ significantly between samples obtained from gastroscopes and colonoscopes, and the difference was approximately 10%. The detection of bacterial colonization (particularly by multiple species) after abdominal surgery is indicative of a complicated and potentially unsafe surgical environment. Contamination with commensal gut bacteria can also lead to pathogenic conditions that may be life threatening [11]. The bacterial profiles of samples cultured from the BCs were diverse with most colonies belonging to a single Gram-negative species (Table 3). In our study, the number of culture-positive samples obtained from colonoscopes was 2-fold higher than those obtained from gastroscopes, and almost 90% of the samples from both endoscopes showed colonization by a single species. Therefore, HLD of GI endoscopes is very important. In terms of the severity of contamination, colonization by multiple species is indicative of greater contamination. In fact, standard reprocessing with HLD is an effective method for decontaminating GI endoscopes according to the current guidelines; 100% decontamination of all GI endoscopes after HLD reprocessing may be impossible, and a culture-positive sample identified as a single species colonization event that is not associated with a clinical outbreak is considered acceptable. In contrast, the bacterial profiles of culture-positive samples obtained from the BCs were diverse, with most samples showing colonization by a single species and most strains were aerobic. Although routine sampling of surfaces within a healthcare facility is generally not recommended by the Centers for Disease Control and Prevention, the Association for the Advancement of Medical Instrumentation, and several healthcare organizations, the practice is recommended by some organizations [12]. We also believed that everything to be too late as clinically required during an outbreak investigation. Dr. Lawrence Muscarella suggested that bacterial growth in a collected sample indicates contamination of the sampled channel, and if no bacterial growth is detected from a surveillance culture this does not necessarily mean that the channel is sterile. Indeed, endoscope sampling is prone to false-negative results and provides data, albeit potentially erroneous data, specific only to the sampled surface [13]. Anyway, our results suggest that culturing samples obtained by rinsing BCs is one of the best methods for performing regular monitoring.

More than 68.4% of the organisms identified were GNGN bacteria, which are associated with a wide range of infections, predominantly those of nosocomial origin. Such infections usually develop in patients with identifiable deficiencies of local and/or systemic immunity. These GNGN bacteria can be isolated from a wide variety of environmental sources, and can cause infection via contaminated medical devices or “pseudoinfections” due to their survival/growth in blood sample tubes or laboratory media. *Pseudomonas aeruginosa* is a GNGN rod-shaped bacterium that is most commonly associated with human infection. Earlier, most species of GNGN bacteria were thought to be contaminants when they were cultured from human specimens, but many have now been shown to be opportunistic pathogens in humans [14]. However, in veterinary medicine, GNGN bacterial species are not considered animal pathogens. Most veterinary microbiology laboratories do not routinely identify GNGN bacteria other than *P*. *aeruginosa*, *Bordetella bronchiseptica*, and *Moraxella bovis*[14-16]. Even our laboratory unit does not routinely identify GNGN bacteria. If GNGN bacterial species are not considered contaminants of AERs after HLD, the percentage of culture-positive rinse samples obtained from the BCs in our study would be reduced to 3.3% (10/300) for gastroscopes and 8.8% (10/120) for colonoscopes. Furthermore, we believe that the primary source of these GNGN is the patient. Although this would suggest that the endoscope cleaning was ineffective, no clinical outbreak occurred because most of these bacteria are not of clinical significance. According to the British Society of Gastroenterology guidelines (February 2008), manual brushing is emphasized for endoscope cleaning and disinfection. If manual brushing was performed correctly, the complete reprocessing cycle after HLD would have been excellent. Rinsing and drying after HLD are essential for the removal of chemical solutions and for preventing bacterial colonization during storage of GI endoscopes [1]. In our study, 42.8% (3/7) of the culture-positive swab samples obtained from AERs showed fungal contamination. This finding highlights the importance of daily forced-air drying of AERs [17]. Therefore, surveillance culturing for both GI endoscopes and AERs is an effective means of monitoring the effectiveness of HLD of GI endoscopes after manual pre-cleaning and decontamination by AER. AERs are effective for decontamination of the outer surfaces of GI endoscopes; however, manual pre-cleaning of all working channels is essential for decontamination of the internal surfaces and should be a high priority.

For endoscope sampling, the BCs were flushed with 50 ml of sterile distilled water, which is an appropriate irrigation solution for bacterial culture in case of the obstruction of external biliary drainage or urinary bladder tube in the general practice. It is a very simple sampling for bacterial culture and also has vigorous results in our previous study [2,8] before ISO 11737–1:2006 documentation. According to the data published, the absence of neutralizing agent in the sampling solution may lead to an underestimation of endoscope contamination levels [18,19]. Both of the references were the experimental contamination and design for the microbiological testing of the sampling solutions. Of course, the results are provided a higher culture-positive rate. Indeed, the sterile distill water is used to washout the possible contaminated material from the BC of the GI endoscopy. The important consideration is the culture agar as blood agar, MacConkey agar, and Lowenstein–Jensen medium in our studies. According to ISO 11737–1:2006 for the sterilization of medical devices, both of the content including determination of a population of microorganisms on product and tests of sterility performed in the validation of a sterilization process was not determined as our practical culture method which was designed before the documentation of ISO 11737–1 and ISO 15883–4. For AER sampling, we swabbed the surface of the AER chamber. This method is not the method recommended in ISO 15883–4 for AER sampling and is not accurate enough. ISO 15883–4 is provided for washer disinfection and emphasized that the methods, instrumentation and instructions required for type testing, works testing, validation (installation, operational and performance qualification on first installation), routine control and monitoring and re-validation, periodically and after essential repairs. We also reported the limitation in our recent reported [17]. The AER sampling result should be used to describe the AER with a high-level disinfection process is enough or not. Therefore, we provided this monitoring method both of the BC sampling and AER swab culture for the patient safety in re-usable medical devices. For the patient safety, regular monitoring the medical devices in daily medical used is very important and a best way to avoid infectious disease outbreak in the hospital. Further clinical studies are warranted to further evaluate our findings as there are no publications documenting any increased risk of infection transmission for endoscopes processed using glutaraldehyde as the HLD.

## Conclusion

In conclusion, culturing samples from GI endoscopes is more effective for monitoring the effectiveness of reprocessing after HLD than culturing swab samples from AERs. Our data suggest that culturing rinse samples from BCs can better indicate the effectiveness of decontamination of GI endoscopes after HLD than culturing swab samples from AERs, which can only indicate whether AERs are free from microbial contamination.

## Competing interests

The authors declare that we have no competing interests. Over the past 5 years, we have not received any reimbursements, fees, funding, or salaries from any organization that may in any way gain or lose financially from the publication of this manuscript either now or in the future. We do not hold any stocks or shares in an organization that may in any way gain or lose financially from the publication of this manuscript either now or in the future. We do not hold and are not currently applying for any patents relating to the content of the manuscript. We have not received reimbursements, fees, funding, or salaries from any organization that holds or has applied for patents relating to the content of this manuscript. There are no non financial competing interests, including political, personal, religious, academic, ideological, intellectual and commercial interests to declare in relation to this manuscript.

## Authors’ contributions

KWC designed the study, and participated in writing the manuscript. MCT performed the data collection and drafted the manuscript. KLW and YCC cultured the bacteria from BCs and AERs samples. MTL was involved in the design of the study and also performed the statistical analyses. THH conceived the study and participated in its design and coordination. All authors read and approved the final manuscript.

## Pre-publication history

The pre-publication history for this paper can be accessed here:

http://www.biomedcentral.com/1471-230X/12/120/prepub
